# Opportunities and Challenges in community-based inclusive science education

**DOI:** 10.1093/eurpub/ckac131.512

**Published:** 2022-10-25

**Authors:** S Scheer, O Orban

**Affiliations:** 1 Health and Social Science, IB University of Applied Health and Social Science, Berlin, Germany; 2 Medical Faculty Department of Health Sciences, Lund University, Lund, Sweden

## Abstract

**Background:**

This research is part of a Horizon2020 funded project named C4S - Community for sciences. The project aims to implement co-created inclusive science activities with children 0-16 years and their families in vulnerable communities. Best practice strategies will be developed to promote the empowerment of children in vulnerable situations. In six European cities, community-based inclusive science activities will be realized and evaluated. What is the impact of inclusive science education on vulnerable communities' health in Europe? Why is community-based inclusive science education an urgent issue to improve health conditions of children and families?

**Methods:**

Guided by the research questions this method includes an integrative literature review to explore barriers and facilitators to delivering inclusive science in European countries and a questionnaire delivered to 6 Hubs involved in the C4S project. The questionnaire identifies core elements of a community, e.g. “Joint action”, “social ties” and “Diversity”. Both the literature review and the community questionnaire are performed in co-creation with inclusive science activities.

**Results:**

Preliminary findings of 214 bibliographical references show the importance and evidence of inclusive science education. When it comes to specific groups of people who are in vulnerable situations, only a few references could be identified. Most results are related to children with disabilities in comparison to children from ethnic minorities. However, ongoing discussions about intersectionality and decolonial theories were identified topics to answer our research questions.

**Conclusions:**

The literature shows evidence about inclusive science but lack of specific information for specific target groups. Therefore, the questionnaire distributed to the Hubs may offer new perspectives and will lead to specific knowledge about communities' needs and resources to ensure inclusive science with and for children in vulnerable situations.

**Key messages:**

• Inclusive and community-oriented science education is relevant to empower children in vulnerable situations.

• New perspectives and deeper knowledge about context specific factors that facilitate inclusive science education may contribute to sustainable health.

